# Transcriptome Sequencing Reveals the Differentially Expressed lncRNAs and mRNAs Involved in Cryoinjuries in Frozen-Thawed Giant Panda (*Ailuropoda melanoleuca*) Sperm

**DOI:** 10.3390/ijms19103066

**Published:** 2018-10-08

**Authors:** Ming-Xia Ran, Yuan Li, Yan Zhang, Kai Liang, Ying-Nan Ren, Ming Zhang, Guang-Bin Zhou, Ying-Min Zhou, Kai Wu, Cheng-Dong Wang, Yan Huang, Bo Luo, Izhar Hyder Qazi, He-Min Zhang, Chang-Jun Zeng

**Affiliations:** 1 College of Animal Sciences and Technology, Sichuan Agricultural University, Chengdu 611130, Sichuan, China; 18227585649@163.com (M.-X.R.); LY13072840231@163.com (Y.L.); yanzhang@sicau.edu.cn (Y.Z.); sicau-liangkai@hotmail.com (K.L.); J1450544201@163.com (Y.-N.R.); zhm3000@126.com (M.Z.); zguangbin@sicau.edu.cn (G.-B.Z.); vetdr_izhar@yahoo.com (I.H.Q.); 2 China Conservation and Research Center for the Giant Panda, Wolong 473000, China; zhouyingmin3552@hotmail.com (Y.-M.Z.); Woki1213@163.com (K.W.); wonsir@gmail.com (C.-D.W.); pandayard@hotmail.com (Y.H.); boluo911@126.com (B.L.); hmhm_zhang@163.com (H.-M.Z.); 3 Department of Veterinary Anatomy & Histology, Faculty of Bio-Sciences, Shaheed Benazir Bhutto University of Veterinary and Animal Sciences, Sakrand 67210, Pakistan

**Keywords:** giant panda, lncRNA, mRNA, frozen-thawed sperm, transcriptome sequencing

## Abstract

Sperm cryopreservation and artificial insemination are important methods for giant panda breeding and preservation of extant genetic diversity. Lower conception rates limit the use of artificial insemination with frozen-thawed giant panda sperm, due to the lack of understanding of the cryodamaging or cryoinjuring mechanisms in cryopreservation. Long non-coding RNAs (lncRNAs) are involved in regulating spermatogenesis. However, their roles during cryopreservation remain largely unexplored. Therefore, this study aimed to identify differentially expressed lncRNAs and mRNAs associated with cryodamage or freeze tolerance in frozen-thawed sperm through high throughput sequencing. A total of 61.05 Gb clean reads and 22,774 lncRNA transcripts were obtained. From the sequencing results, 1477 significantly up-regulated and 1,396 significantly down-regulated lncRNA transcripts from fresh and frozen-thawed sperm of giant panda were identified. GO and KEGG showed that the significantly dysregulated lncRNAs and mRNAs were mainly involved in regulating responses to cold stress and apoptosis, such as the integral component of membrane, calcium transport, and various signaling pathways including PI3K-Akt, p53 and cAMP. Our work is the first systematic profiling of lncRNA and mRNA in fresh and frozen-thawed giant panda sperm, and provides valuableinsights into the potential mechanism of cryodamage in sperm.

## 1. Introduction

The giant panda (*Ailuropoda melanoleuca*) is an endangered species confined to south-central China. Natural mating and artificial insemination are common approaches for breeding of the giant panda in captivity. Artificial insemination has been proven to play an important role in assisted reproduction in humans and other mammals. However, the artificial insemination of giant pandas mainly employs fresh sperm, while frozen-thawed sperm is rarely used in this procedure. The lower farrowing rate also explains the poor utilization efficiency of giant panda frozen-thawed sperm. Furthermore, only about 25% (3/11) of giant pandas at the ideal breeding age are mating naturally [1]. The genetic management of captive giant panda is recognized as one of the highest priorities of ex-situ conservation action in China. The ability to consistently produce offspring using cryopreserved sperm would make substantial improvement to giant panda breeding as cryopreserved sperm can be stored for a longer time [2]. It has been reported that more than 30% of frozen-thawed sperm can be utilized for artificial insemination with successful conception and birth of giant panda [3]. For example, 4 out of 7 giant pandas were pregnant after artificial insemination performed by Huang and co-authors, yielding a breeding success rate of 57.1% [4].

Substantial efforts have also been made on the selection and screening of cryoprotectants, antioxidants and freeze-thawing programs [5,6,7,8]. Through exploring different freezing diluents (TEST sucrose, egg yolk and glycerol), freezing methods, and thawing solutions, a new procedure for preparing 0.25 mL frozen panda semen was established [5]. Giant panda sperm appears to be strongly cryo-resistant and can survive repeated cycles of freezing-thawing [9]. However, the pregnancy rate of insemination using frozen-thawed semen was only 28.57%, which is less than the 33.3% observed in insemination using fresh semen and in natural mating [3]. A loss in sperm motility immediately after thawing was observed compared with the pre-freeze motility in giant panda sperm. More sperms were capacitated than fresh sperms after the freezing procedure [2]. However, the mechanism of freeze-tolerance and cryoinjury in giant panda sperm remains unclear.

Mature sperm cells contain RNA [10], and might retain certain transcriptional and translational activities [11,12]. Gur and Breitbart have demonstrated that protein expression from nuclear genes does, in fact, occur in sperm. Both mRNAs and their translated proteins were observed to be localized inside and outside of the mitochondria [13,14]. It has also been shown that ejaculated sperms can translate protein from mRNA transcripts during the final maturation steps prior to fertilization. Besides, knocking down the hyperactivation-associated mRNA by transfecting siRNA can inhibit the level of cAMP and protein oxidative phosphorylation in spermatozoa and reduce the level of sperm hyperactivation [15,16]. Recently, microRNA (miRNA) and long non-coding RNA (lncRNA) have been demonstrated to be involved in spermatogenesis [17], sperm cryoinjuries [18] and fertility [19]. The non-coding RNA with length greater than 200 nucleotides is defined as lncRNA [20]. Numerous evidences have shown that lncRNA is a novel regulatory gene that plays important roles in cell development, pluripotency, cell growth and apoptosis [21,22,23,24,25]. Furthermore, lncRNA is also crucial in the regulation of sperm function. As illustrated by microarray analysis, lncRNA is regulated dynamically and is expressed mainly in meiosis and haploid stages during spermatogenesis [26]. The survival rate of spermatogonial stem cell was significantly decreased when *lncRNA033862* was knocked out [27]. In addition, apoptosis of spermatocytesin pachytene was increased after Tsx knockout [28]. Furthermore, lncRNA *HOTAIR* could increase the activity of superoxide dismutase (SOD) in human sperm by enhancing *Nrf2* expression, which could ultimately affect sperm quality [29]; Over-expression of *mil-HongrES2*, sheared from *HongrES2*, could affect sperm capacitation by inhibiting the expression of *CES7* [30]. Differential expressions of lncRNA and mRNA between diabetic and normal sperm, along with its role in the diabetes-related low fertility, were also uncovered by high throughput sequencing and lncRNA–mRNA interaction studies [31].

To date, the contribution of lncRNA and mRNA in the regulation of cold response in cryopreserved giant panda sperm has yet to be elucidated. Here, we employed a high throughput sequencing approach to explore the expression profiles of mRNA and lncRNAs in fresh and frozen-thawed giant panda sperm, with the goal to better understand the potential role of differentially expression of lncRNAs and mRNA in sperm cryoinjury or cryodamage during cryopreservation.

## 2. Result

### 2.1. Sperm Quality before and after Cryopreservation

The average volume of electro-ejaculation was 2.50 ± 0.35 mL with concentration of 16.71 ± 4.36 × 10^8^ mL^−1^. The sperm motility was significantly decreased from 0.83 ± 0.08 to 0.63 ± 0.10 before and after cryopreservation, respectively (*p* < 0.05).

### 2.2. RNA Quality Inspection

**Figure 1:**
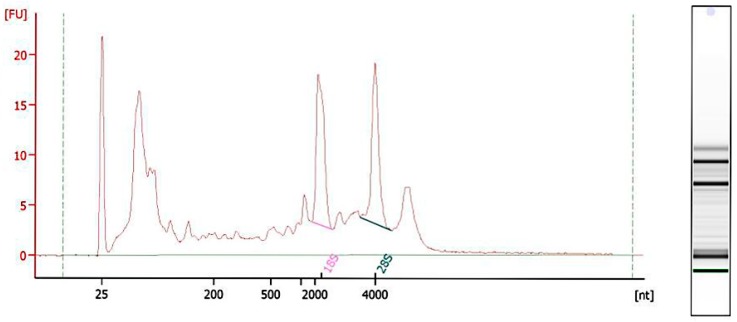
RNA integrity analyzes of giant panda sperm showed 28S and 18S.

RNA integrity was assessed using the RNA Nano 6000 Assay Kit of the Agilent Bioanalyzer 2100 System (Agilent Technologies, Santa Clara, CA, USA) (Figure 1).

### 2.3. RNA Sequencing Roundup

After sequencing quality control, we obtained 61.05 Gb of clean data, and the Q30 base percentages of each sample were no less than 89.25%. The mapping rate of blasted fresh and frozen-thawed sperm to the latest giant panda reference genome were 46.30% and 57.78%, respectively.

### 2.4. Identification of lncRNAs and mRNA

**Figure 2:**
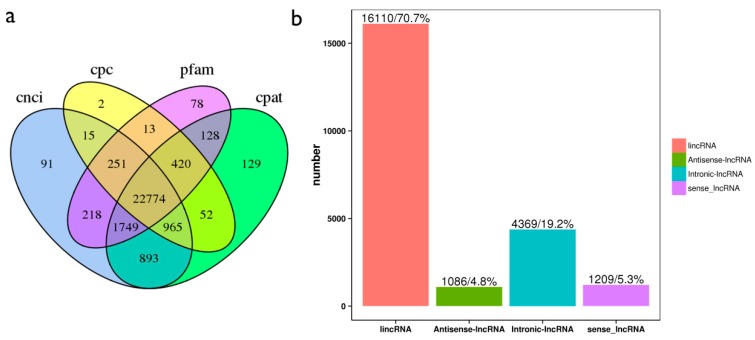
(**a**) Coding potential analysis of Venn diagram. Four tools (CNCI, CPC, CPAT and Pfam-scan) were selected to analyze the coding potential of lncRNAs. The data shared by the four tools were designated as candidates for subsequent analyses. (**b**) The identified lncRNAs were divided into four types, including intergenic lncRNA, antisense lncRNA, sense lncRNA and intronic lncRNA, and the number and proportion of each type of lncRNAs were also calculated.

The qualified transcripts were analyzed using the CNCI, CPC and Pfam-scan software. We identified a total of 22,774 lncRNAs (Figure 2a), among which 16,110 of them were lincRNAs including 1086 antisense lncRNAs, 4369 intronic lncRNAs, and 1209 sense lncRNA (Figure 2b). In addition, 32,322 protein-coding transcripts were also identified, which contains 13,186 new genes (Tables S1 and S2).

### 2.5. Characteristic Comparison of lncRNAs and mRNAs

**Figure 3:**
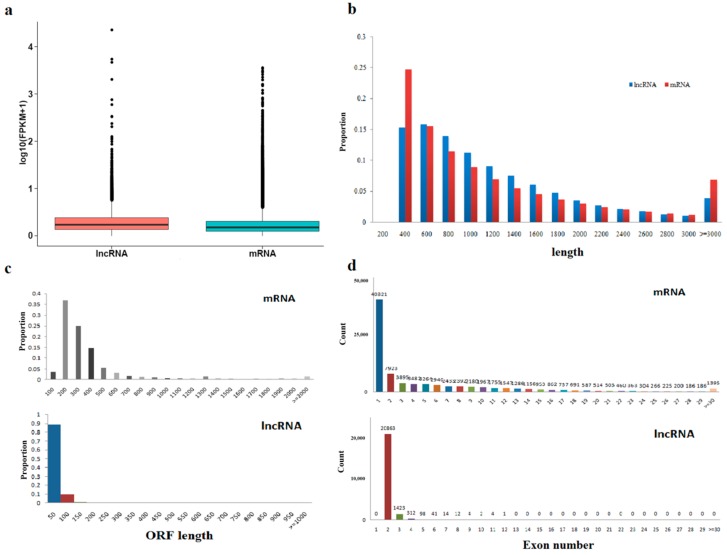
Comparison of the identified lncRNAs and mRNAs. (**a**) Expression level analysis of the mRNAs and lncRNAs. (**b**) The length distribution of lncRNAs and mRNAs. The abscissa represents length, and the ordinate is the number of RNA with length in this range. (**c**) Distribution of open reading frame lengths (ORF) in the mRNAs and lncRNAs. The abscissa represents ORF length, and the ordinate is exon numbers distributed in the range of RNA numbers. (**d**) Exon number distribution of lncRNAs and coding transcripts, the abscissa is exon numbers, and the ordinate is exon numbers distributed in the range of RNA numbers.

Expression of lncRNA was higher than that of messenger RNA, mRNA. However, the average length and open reading frame (ORF) length of mRNA were longer than those of lncRNA (Figure 3a–c). Moreover, less lncRNA were identified compared to mRNA based on the number of exons sequenced (Figure 3d).

### 2.6. Differential Expression Analysis

**Figure 4:**
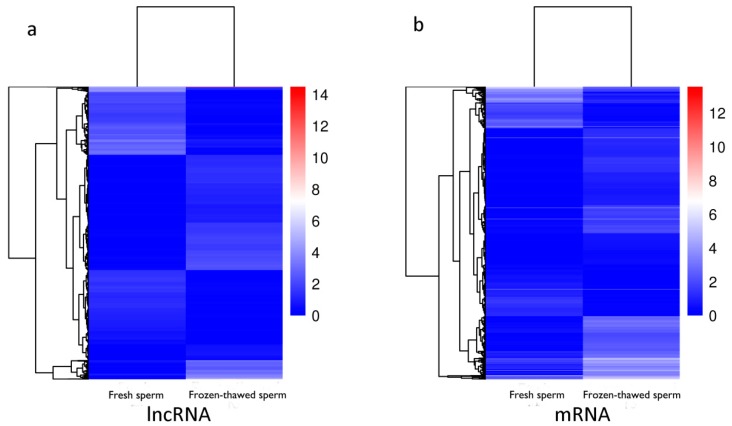
The heat maps of Cluster analysis of differentially expressed lncRNAs and mRNAs. (**a**) lncRNAs, (**b**) mRNAs. Red color indicated an increase in expression, and blue color indicated a decrease in expression.

Fold Change ≥2.0 and FDR <0.05 were used as screening criteria. A total of 2873 lncRNAs were differentially expressed between fresh and frozen-thawed sperm, among which 1477 lncRNAs were up-regulated and 1396 lncRNAs were down-regulated (Table S3, *p* < 0.05). Results from cluster analysis of differentially expressed lncRNAs are presented as a heat map (Figure 4a). Meanwhile, 5226 significantly dysregulated mRNA transcripts were also identified, among which 3581 mRNAs were up-regulated and 1645 mRNAs were down-regulated in frozen-thawed sperm (Table S4, *p* < 0.05). Results from cluster analysis of differentially expressed mRNAs are shown in a heat map (Figure 4b).

### 2.7. Target Genes Prediction of cis- and trans-LncRNAs

LncRNAs can act on target genes, either in *cis* or in *trans* to co-expression with target genes. In order to explore the possible functions of differentially expressed lncRNAs, the target genes of lncRNA were predicated using the *cis* and *trans* model. The results showed that 7689 lncRNAs have the predicted *cis* target gene, and 1333 lncRNAs have the predicted *trans* target gene (Tables S5 and S6).

### 2.8. qRT-PCR Validation

**Figure 5:**
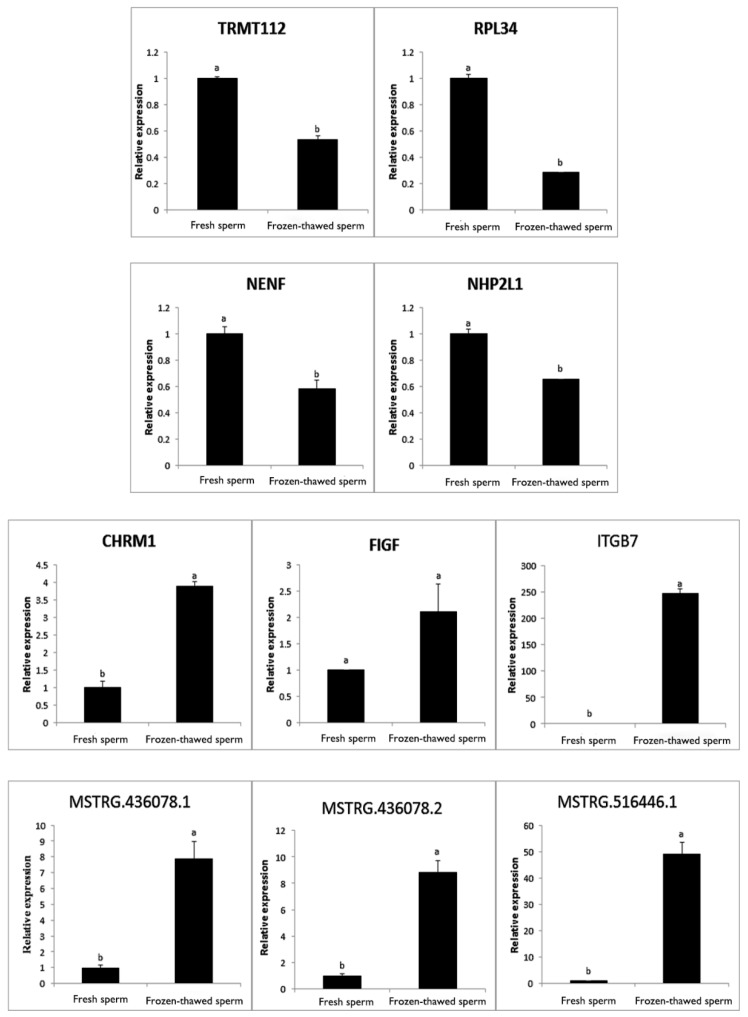
Validation of transcript expression by qRT-PCR. Glyceraldehyde-3-phosphate dehydrogenase (GAPDH) gene was used as a housekeeping internal control. Transcript expression was quantified relative to the expression level of GAPDH using the comparative cycle threshold (2^−ΔΔ*C*t^) method. Different letter indicates *p* < 0.05.

Three lncRNAs and seven mRNAs that were differentially expressed between fresh and frozen-thawed sperm were selected for data validation of high throughput sequencing using qRT-PCR. Validation showed that all results were consistent with RNA-seq data, except for one mRNA that was not significantly differentially expressed (*p* < 0.05) (Figure 5). The result indicated that the expression levels of all lncRNAs and mRNA were consistent with RNA-seq data, which confirmed the reliability of RNA-seq and laid a solid foundation for further exploration.

### 2.9. Functional Enrichment Analysis of GO and KEGG

GO analysis of predicted *cis*-lncRNA targets demonstrated 202 significantly enriched terms (Table S7, *p* < 0.05). The top 5 terms were involved in single-organism process (GO: 0044699), olfactory receptor activity (GO: 0004984), detection of chemical stimulus involved in sensory perception of smell (GO: 0050911), G-protein coupled receptor activity (GO: 0004930), and signal transducer activity (GO: 0004930). Interestingly, the trans-membrane signaling receptor activity (GO: 0004888), membrane (GO: 0016020) and mitochondria (GO: 0005739) associated with sperm physiological and structural changes, were also significantly enriched in frozen-thawed sperm. KEGG analysis of the lncRNAs targets showed 10 enriched terms (Table S8, *p* < 0.05). A number of target genes were annotated to the Olfactory transduction, apoptotic-associated pathway, including JAK-STAT (ko04142), Calcium (ko04020), and PI3K-Akt signaling pathway (ko04151).

Among the *trans*-lncRNA target genes, 233 GO terms were significantly enriched (Table S9, *p* < 0.05). The top 5 terms were involved in cell development (GO: 0048468), Ral GTPase activator activity (GO: 0005096), metanephric loop of Henle development (GO: 0072236), metanephric distal tubule development (GO: 0072235) and activation of Ral GTPase activity (GO: 0090630). Similar to the results of *cis*-lncRNA target genes, sperm membrane-related terms were significantly enriched, including membrane (GO: 0016020), integral component of membrane (GO: 0016021), and plasma membrane (GO: 0005886). Besides, 6 significantly enriched KEGG pathways were detected (Table S10, *p* < 0.05). Furthermore, 3 apoptotic-related pathways, calcium, p53 and PI3K-AKT signaling pathways were also involved.

GO and KEGG analyses of 5,226 significantly dysregulated mRNAs showed that 365 GO terms (Table S11, *p* < 0.05) and 14 significantly enriched pathways (Table S12, *p* < 0.05) were highly enriched. Similar to the results of lncRNAs, a number of genes were annotated to the olfactory transduction, PI3K-AKT signaling pathway, JAK-STAT signaling pathway, Calcium signaling pathway, membrane and the integral component of membrane.

### 2.10. Co-Expression of LncRNAs and mRNA

The results of all lncRNAs and their target mRNAs were simultaneously and significantly differentially expressed in fresh and frozen-thawed sperms, which were summarized in Table S13.

## 3. Discussion

In this study, total RNA was extracted from motile and non-motile giant panda sperm after cryopreservation. Using the entire sperm population is representative of the natural transcript variation [32], and the increase of sperm apoptosis after cryopreservation is a part of sperm cryoinjury [33].

To date, this study is the first systematical lncRNA and mRNA profiling analysis of fresh and frozen-thawed sperms in the giant panda by high throughput sequencing. We acquired a total of 22,774 predicted lncRNAs and 32,322 mRNAs from giant panda sperms. Among 32,321 mRNAs, 13,186 novel genes and 19,136 known genes were identified. In general, the lncRNAs contained fewer exons, exhibited shorter overall length and average open reading frame length, and showed lower expression level than mRNAs, which was consistent with previous reports on goats, mice, pigs and other mammals [34,35,36]. The shared characteristics of lncRNAs in mammals implicate their important roles in the regulation, control, and guidance of sperm function.

Evidences have shown that sperm viability decreased by at least 50% because of cryodamage or cryoinjury during cryopreservation. Generally, sperm cryoinjury includes structural damage and functional changes. The process of cryopreservation could lead to increase in cell membrane fluidity, loss of plasma membrane integrity, impaired membrane protein function, decrease in sperm antioxidant activity, increase in oxidative stress and ROS levels, oxidative damage to DNA, mitochondrial damages, decrease in membrane potential, and altered phosphatidylserine reversion [37,38,39,40]. Among the adverse effects of cryopreservation, DNA oxidative damage, decrease in mitochondrial membrane potential and phosphatidylserine externalization are the main physiological characteristics of sperm apoptosis. In addition, it is believed that the cryopreservation process induces capacitation-like changes to sperm. Some of the similar changes observed between in vitro capacitation and cryo-capacitation include plasma membrane reorganization, increase in intracellular Ca^2+^ concentration and occurrence of PTP [41,42,43]. In this study, we identified 2873 lncRNAs and 5226 mRNAs that were significant differentially expressed between fresh and frozen-thawed sperm. The mRNAs targeted by these lncRNAs and the differentially expressed mRNA were mainly enriched in membrane-related terms (integral component of membrane and membrane) and responses to stimulus. Similar GO enrichment results were observed in GO analysis of differentially expressed proteins in rainbow trout frozen sperm [44] and differentially expressed miRNAs of porcine frozen sperm [18]. Moreover, these membrane-related terms could be associated with sperm cryodamage. In fact, KEGG analysis showed that mRNA, lncRNAs’ *cis*-target genes and miRNAs’ target genes were most widely distributed in the olfactory factor transduction pathway associated with membrane depolarization. The increase of membrane depolarized sperm isrelated to the apoptosis, and it is one of the reasons for the low fertilization rate of frozen thawed sperm [45]. It suggests that depolarization of sperm membrane associated with cAMP may be an important change in sperm membrane during cryopreservation. Furthermore, many lncRNA target genes or differentially expressed mRNAs were enriched in the PI3K-Akt, p53, Calcium, cAMP and MAPK signaling pathways. Among them, PI3K-Akt, p53, and Calcium signaling pathways were apoptosis-related pathways. Similar results from analysis of cryopreserved bull sperm also indicated target mRNAs of miRNAs and piRNAs were mainly involved in apoptotic-related pathways, especially in PI3K-Akt pathway [46]. In addition, cAMP and MAPK signaling pathways were capacitation-related pathways. These results support the conclusion that capacitation-like changes are induced during the process of sperm cryopreservation [47].

It is widely accepted that mature sperm cells contain RNA. These RNA molecules are thought to be remnants of transcription during spermatogenesis [10]. Gur and Breitbart [13] have demonstrated that protein transcription from nuclear genes occurs in sperm. It has also been shown that protein translation from mRNA transcripts takes place in ejaculated sperm during the final maturation steps prior to fertilization [14]. Furthermore, lncRNA also participates in the regulation of sperm capacitation [30], spermatogenesis [17] and definition of sperm parameters [19]. In the present study, both lncRNAs and their target genes were significantly dysregulated between fresh and frozen-thawed giant panda sperm (Table S11). Among them, 13 lncRNAs and 11 of their target mRNAs were found to be associated with sperm fertilization, spermatogenesis, and sperm capacitation or acrosome reaction (Table 1). In addition, 22 differentially expressed mRNAs were involved in sperm apoptosis (8 anti-apoptotic and 14 pro-apoptotic) (Table 2). Specifically, 4 mRNAs (*RHOA*, *CDK5*, *MTA1*, and *CACNA1G*) were involved in apoptosis and sperm function. In addition, lncRNA *MSTRG.531884.1* and its 3 target mRNAs (*CCL24*, *RHOBTB2*, and *CACNA1G*) were differentially expressed and were associated with sperm apoptosis. Meanwhile, lncRNA *MSTRG.655028.1* could regulate another 2 apoptotic-related differentially expressed mRNAs, *MTA1* and *CRIP2*. A total of 4 mRNAs (*FGFR4*, *SESN2*, *CRLF2* and *CACNA1G*) were annotated to apoptotic-related PI3K-Akt, p53, JAK-STAT, and Calcium signaling pathway, respectively. Therefore, we concluded that these differentially expressed lncRNAs and mRNAs may be involved in the regulatory roles of apoptosis during sperm cryopreservation. Generally, the function of lncRNAs is reflected by their effects on protein-coding genes. Many kinds of regulatory mechanisms between lncRNA and mRNA have been reported, including the guiding, combinational [48,49], protective [50], and competitive relationships that ultimately lead to inhibition of gene transcription, and promotion or inhibition of mRNA degradation, and finally, regulation of the expression level of mRNA. We speculated that lncRNA may also be involved in the regulation of cold response, freeze tolerance or cryoinjuries during sperm cryopreservation. However, the mechanism of regulation during sperm cryopreservation by interaction of differentially expressed lncRNAs and mRNAs is still unclear and warrants further investigation.

## 4. Materials and Methods

### 4.1. Animal Ethics Statement

Semen collection and treatment were conducted according to the Regulations of the Administration of Affairs Concerning Experimental Animals (Ministry of Science and Technology, China, revised in June 2004) and approved by the Institutional Animal Care and Use Committee in the College of Animal Science and Technology, Sichuan Agricultural University, Sichuan, China, under permit No. DKYB20151013 (13 October 2015). Furthermore, all experimental protocols were approved by the College of Animal Science and Technology, Sichuan Agricultural University (NO. DKYB20151013, 13 October 2015).

### 4.2. Sperm Collection and Cryopreservation

Giant pandas (*n* = 5) that were disease-free and exhibited normal fertility, sexual maturity and normal semen quality were selected from the Bifengxia base of China Conservation and Research Center for the Giant Panda. Semen was collected using an electro-ejaculation method [88]. Then, semen was kept in 37 °C water bath, and SQA-V semen quality analyzer (MES, Caesarea Industrial Park, Israel) was used to evaluate sperm quality parameters according to previous report [89]. The semen of 5 giant pandas was pooled then equally divided into two groups (Fresh sperm and cryopreserved sperm). The fresh semen was directly used for RNA extraction. Then, another aliquot of the semen was mixed in TEST-yolk buffer (TYB) with glycerol & gentamicin frozen diluents (Irvine Scientific, Santa Ana, CA, USA) according to the manufacturer’s instruction. Freezing protocols were performed according to the methods described in Spinder et al. [2]. In brief, sperms were diluted in TEST egg yolk buffer (Irvine Scientific) then combined with glycerol (final concentration of 5% glycerol). Then, sperms were load into 0.25 mL straws and slowly cooled to 4 °C in a refrigerator over 4 h and then placed above liquid nitrogen (LN) to equilibrate at a rapid cryopreservation rate of −40 °C /min (at 7.5 cm above LN for 1 min) and −100 °C /min (at 2.5 cm above LN for 1 min). Finally, all straws were submerged and storage in LN until use. During thawing, the straws were immersed into a 37 °C water bath for 30 s and diluted with equal volume of HF10 (Ham’s F10 medium with 5% fetal calf serum and 25 mM HEPES).

### 4.3. Total RNA Extraction, Library Preparation, and Sequencing

Before total RNA extraction, semen was washed three times to remove seminal plasma. The, sperm was treated with 0.5% of Triton X-100 to avoid somatic cells contamination according to previous study from our lab [90]. Then, total RNA was extracted from each sample according to the instruction manual of the TRIzol LS reagent (Invitrogen, Carlsbad, CA, USA). RNA degradation and contamination, especially DNA contamination, was monitored on 1.5% agarose gels. RNA concentration and purity were measured using the NanoDrop 2000 Spectrophotometer (Thermo Fisher Scientific, Wilmington, DE, USA). RNA integrity was assessed using the RNA Nano 6000 Assay Kit of the Agilent Bioanalyzer 2100 System (Agilent Technologies, Santa Clara, CA, USA). A total amount of 1.5 μg RNA per sample was used as input material for rRNA removal using the Ribo-Zero rRNA Removal Kit (Epicentre, Madison, WI, USA). 

Sequencing libraries were generated using NEB NextR Ultra Directional RNA Library Prep Kit for Illumina (NEB, Ipswich, MA, USA) following manufacturer’s recommendations and index codes were added to attribute sequences to each sample. In order to select insert fragments of preferentially 150–200 bp in length, the library fragments were purified with AMPure XP Beads (Beckman Coulter, Beverly, MA, USA). Then PCR was performed with Phusion High-Fidelity DNA polymerase, Universal PCR primers and Index (X) Primer. At last, PCR products were purified (AMPure XP system) and library quality was assessed on the Agilent Bioanalyzer 2100 and qPCR, and then sequenced by Illumina Hiseq 2000 platform.

### 4.4. Quality Analysis, Mapping, and Transcriptome Assembly

Clean data (clean reads) were obtained by removing reads that contained adapter, and ploy-N and of low quality from raw data. All the downstream analyses were based on clean data of high quality. Sequence alignment and subsequent analysis were performed using designated reference genome of giant panda (*Ailuropoda melanoleuca*) (available online: http://ftp.ncbi.nlm.nih.gov/genomes/all/GCF/000/004/335/GCF_000004335.2_AilMel_1.0). Then, clean reads were mapped to the giant panda (*Ailuropoda melanoleuca*) genome sequence with HISAT2 [91]. The mapped reads of each sample were assembled by StringTie [92].

### 4.5. Identification and Expression Analysis of lncRNA and mRNA

The transcriptome was assembled based on the reads mapped to the reference genome. StringTie was used to calculate the read coverage of each transcript, and those with less than three read coverage were removed. Furthermore, tRNA, rRNA, snoRNA, snRNA, pre-miRNA, and pseudo-genes were also discarded. Then, the assembled transcripts were annotated using the gffcompare program. The qualified lncRNAs were immediately classified as known lncRNAs. The unknown transcripts were used to screen for putative lncRNAs. The unknown transcripts with lengths longer than 200 nt and have more than two exons were selected as lncRNA candidates and subjected to further screening using CPC/CNCI/Pfam. The different types of lncRNAs, including lincRNA, intronic lncRNA, and anti-sense lncRNA, were selected using cuffcompare. Fragments per kilo-base of exon per million fragments mapped (FPKMs) of both lncRNAs and coding genes in each sample were calculate by StringTie (v1.3.1). Gene FPKMs were computed by summing the FPKMs of transcripts in each gene group based on the length of the fragments and read count mapped to that fragment.

Based on the selected reference genome sequence, the Cufflinks (v2.2.0) software was used to splice the Mapped Reads and compare with the original annotation information to find the original unannotated transcriptional area and explore the new transcriptional and new genes of the species, so as to supplement and improve the original annotation information of the original group. Filter out the short (less than 50 amino acid residues) of the encoded peptide chain or contain only a single exon sequence.

### 4.6. Differential Expression Analysis

Differential expression analysis of the two groups was performed using the DESeq R package (1.10.1). Genes with an adjusted *p* < 0.01 and absolute value of log2 (Fold change) >1 were assigned as differentially expressed. Differential expression analysis of two samples without biological replicates was performed using the EBseq (2010) R package, and *q*-value < 0.01 & |log2 (fold change)|>1 were set as the threshold for significant differential expression.

### 4.7. Target Gene Prediction

In this study, *cis*- and *trans*-analyses were used to predict the target genes of lncRNAs. Briefly, the coding genes that were 100 K upstream and downstream of lncRNAs were searched as *cis* results. LncTar target gene prediction tools were used to predict *trans*-target genes of lncRNA.

### 4.8. qRT-PCR Validation

qRT-PCR was performed using SYBR Premix Ex Taq II (TaRaKa Biotech, Dalian, China) on a StepOnePlus real-time PCR system (Applied BioSystems, Foster City, CA, USA) using an annealing temperature of 60 °C according to our laboratory’s protocols. The specific quantitative primers for 10 transcripts were listed in Table 3. In addition, GAPDH was used as an endogenous control. The conditions were as follows: 95 °C for 30 s, followed by 40 cycles (95 °C for 5 s and 60 °C for 30 s; next, 95 °C for 10 s, 60 °C for 2 s; Finally, 60 to 95 °C, increment 0.5 °C for 2 s). Each experiment was performed intriplicate.

### 4.9. GO and KEGG Enrichment Analyses

GO enrichment analysis was applied to target genes of lncRNAs using the GOseqR package. In addition, the differentially expressed protein coding genes were also analyzed using GO. The enrichment of lncRNA target genes or differentially expressed protein-coding genes in KEGG pathways were analyzed by the KOBAS (v3.0, Center for Bioinformatics, Peking University, China) software.

### 4.10. Statistical Analysis

The statistical differences were analyzed using the SPSS (version 20.0, IBM, Chicago, IL, USA) by independent-samples *t*-test. All data were shown as the means ± SEM. *p* values < 0.05 were regarded as statistically significant.

## 5. Conclusions

In conclusion, our work is the first to provide the expression profiles of lncRNAs, and mRNAs in fresh and frozen-thawed giant panda sperm. These differentially expressed lncRNAs and mRNAs are found to be involved in the function of sperm membrane, metabolism, capacitation, apoptosis, and definition of post-thawed sperm quality parameters. Our findings provide valuable insights for future investigation of the mechanism of sperm cryoinjury and freeze tolerance during cryopreservation. 

## Figures and Tables

**Table 1:** Function of lncRNAs and target mRNAs that were significantly differentially expressed between fresh and frozen-thawed giant panda sperm. Log2FC, log2 fold-change.

lncRNA	log2FC	Target mRNA	log2FC	Description
*MSTRG.122368.1*	3.14	*CDK5*	2.76	Regulating of sperm tail development [51]
*MSTRG.655028.1*	−2.86	*MTA1*	−3.33	Crucial for spermatogenesis [52]
*MSTRG.332212.2*	3.86	*RHOA*	−3.02	Involved in capacitation and the acrosome reaction [53]
*MSTRG.531884.1*	3.93	*CACNA1G*(Cav3.1)	2.74	Regulate male fertility in mice [54]
*MSTRG.264076.1*	3.81	*SPAG8*	3.18	Cell division during spermatogenesis [55]
*MSTRG.480606.2*	4.12	*SPEM1*	-2.53	Spermatogenesis-essential proteins [56]
*MSTRG.408558.1*	3.43	*TPST2*	3.01	Male infertility, sperm motility defects [57]
*MSTRG.369918.1*	5.54	*PRSS37*	−3.28	Prss37 deletion markedly decreased fertilization rate [58,59]
*MSTRG.1467.1*	2.81	*CNGB1*	4.88	Differentially represented between smokers and non-smokers’ sperm [60]
*MSTRG.169617.1*	4.46	*CATSPER1*	−2.91	Involved in hyperactivation and essential for fertility [61], functional failure of *CatSper* is sufficient to compromise fertility of human sperm [62]
*MSTRG.597193.1*	3.97	OVGP1	2.66	Sustaining the sperm functions, include motile, membrane intact, proportion of capacitated and acrosome- reacted [63]
*MSTRG.651082.1*	3.38	CCNA1	−3.08	Essential for spermatogenesis in the mouse. Ccna1- deficient spermatocytes arrest at late meiotic prophase and undergo apoptosis [64]
*MSTRG.651082.3*	−3.30			

**Table 2:** Apoptosis-related mRNAs and their corresponding lncRNAs that were significantly differentially expressed between fresh and frozen-thawed giant panda sperm. “−” means anti-apoptosis, “+” means pro-apoptosis. Log2FC: log2 fold-change.

Gene Name	Log2FC	Function	Ref.	lncRNA	Log2FC
*RHOBTB2*	2.87	+	[65]	*MSTRG.531884.1*	3.93
*FGFR4*	3.37	+	[66]	*MSTRG.628795.1*	4.88
*MTA1*	−3.33	−	[67]	*MSTRG.655028.1*	−2.86
*PDCD2*	−2.72	+	[68]	*MSTRG.509816.1*	4.02
*TP53INP1*	−2.91	+	[69]	*MSTRG.561458.1*	−3.23
*CRIP2*	−3.74	+	[70]	*MSTRG.655028.1*	−2.86
*CACNA1G*	2.74	+	[71]	*MSTRG.531884.1*	3.93
*CCL24*	5.23	+	[72]	*MSTRG.444753.1*	4.88
*BAG5*	−2.70	−	[73]	*MSTRG.263206.1*	4.14
*POU4F1*	4.17	+	[74,75]	*MSTRG.306867.1*	−3.32
*S100P*	−2.52	−	[76]	*MSTRG.552283.1*	3.52
*CDK5*	2.76	+	[77]	*MSTRG.122368.1*	3.14
*ETV4*	2.60	+	[78]	*MSTRG.298973.1*	4.73
*PRPS2*	−2.66	−	[79]	*MSTRG.101520.1*	−5.39
				*MSTRG.101521.1*	−4.27
*SESN2*	5.16	+	[80]	*MSTRG.33577.3*	3.73
*TNFSF14 (LIGHT)*	5.66	+	[81]	*MSTRG.609960.1*	−3.08
*PRSS8*	4.58	+	[82]	*MSTRG.493468.1*	4.16
*AHSA1*	−2.66	−	[83]	*MSTRG.555075.1*	5.64
*CYGB*	5.05	−	[84]	*MSTRG.301513.1*	−5.91
*DDIT3* (*CHOP*)	−2.70	+	[85]	*MSTRG.141461.2*	5.18
*RHOA*	−3.02	−	[86]	*MSTRG.332212.2*	3.86
*CRLF2*	3.02	−	[87]	*MSTRG.343934.1*	−4.49

**Table 3:** Primers used for qRT-PCR and validation.

Gene Name	Primer Sequence (5′–3′)	Amplicon (bp)
*ITGB7*	F: AGGTCTCATCCCCCGAGAAG	168
	R: CGTACACAGGGTTCAAAGGC	
*MSTRG.436078.1*	F: CAGGCTTCCTCCTCTCTCCA	143
	R: CCACCAGATCTCAAGGACAGC	
*MSTRG.436078.2*	F: GCCTGTCTCATTGCTCAAGGT	114
	R: GGACTATTCTGGTAGCTGTGTCCA	
*CHRM1*	F: GCAAGTGGCTTTCATTGGGA	133
	R: CAGGCTGAGCAGGAAGTAGT	
*MSTRG.516446.1*	F: GGAGAATTACGGTGGGATGAC	149
	R: AAGAAAACACTAACGCAGAAAGG	
*FIGF(VEGFD)*	F: AAGGAGAAGAGGGCTGCCTA	117
	R: GACAGCAACTTGGCAAAGCA	
*NENF*	F: TGGCAGTGAAAGGAGTGGTGTT	151
	R: CCCGTAGTGTCATGGGTGAGGT	
*RPL34*	F: AAACTAGGCTGTCCCGAACC	119
	R: AGCACGAACTCCTCGAAGTC	
*NHP2L1*	F: CGGAAAGGAGCCAATGAAG	205
	R: CAGAACAGGCGATGACAGG	
*TRMT112*	F: TGGCGCGTATGATACCCAAG	209
	R: GCGACTGATGGGGAACAGAT	

## Supplementary Materials

Supplementary materials can be found at http://www.mdpi.com/1422-0067/19/10/3066/s1.
